# Genome-wide association study identifies genes associated with neuropathy in patients with head and neck cancer

**DOI:** 10.1038/s41598-018-27070-4

**Published:** 2018-06-08

**Authors:** Cielito C. Reyes-Gibby, Jian Wang, Sai-Ching J. Yeung, Patrick Chaftari, Robert K. Yu, Ehab Y. Hanna, Sanjay Shete

**Affiliations:** 1 Department of Emergency Medicine, The University of Texas MD Anderson Cancer Center, Houston, Texas 77030 USA; 2 Department of Biostatistics, The University of Texas MD Anderson Cancer Center, Houston, Texas 77030 USA; 3 Department of Head and Neck Surgery, The University of Texas MD Anderson Cancer Center, Houston, Texas 77030 USA; 4 Department of Epidemiology, The University of Texas MD Anderson Cancer Center, Houston, Texas 77030 USA

## Abstract

Neuropathic pain (NP), defined as pain initiated or caused by a primary lesion or dysfunction in the nervous system, is a debilitating chronic pain condition often resulting from cancer treatment. Among cancer patients, neuropathy during cancer treatment is a predisposing event for NP. To identify genetic variants influencing the development of NP, we conducted a genome-wide association study in 1,043 patients with squamous cell carcinoma of the head and neck, based on 714,494 tagging single-nucleotide polymorphisms (SNPs) (130 cases, 913 controls). About 12.5% of the patients, who previously had cancer treatment, had neuropathy-associated diagnoses, as defined using the ICD-9/ICD-10 codes. We identified four common SNPs representing four genomic regions: 7q22.3 (rs10950641; SNX8; *P* = 3.39 × 10^−14^), 19p13.2 (rs4804217; PCP2; *P* = 2.95 × 10^−9^), 3q27.3 (rs6796803; KNG1; *P* = 6.42 × 10^−9^) and 15q22.2 (rs4775319; RORA; *P* = 1.02 × 10^−8^), suggesting SNX8, PCP2, KNG1 and RORA might be novel target genes for NP in patients with head and neck cancer. Future experimental validation to explore physiological effects of the identified SNPs will provide a better understanding of the biological mechanisms underlying NP and may provide insights into novel therapeutic targets for treatment and management of NP.

## Introduction

Head and neck cancer (HNC) is the sixth-most-common malignancy worldwide. Patients with HNC live longer relative to patients with other cancers. The treatment and management of pain is a significant clinical concern in this population. Pain is a common symptom reported by head and neck cancer patients, resulting from tumor invasion or compression of anatomic structures. Acute pain is extremely common during and after surgery, chemo- and/or radiotherapy. An estimated 80% of head and neck cancer patients report pain during treatment, and pain persists for some 36% of patients beyond treatment^1^. Neuropathic pain (NP), defined as pain initiated or caused by a primary lesion or dysfunction in the nervous system, is a debilitating chronic pain condition often resulting from cancer treatment. Patients typically describe it as a burning, shooting, or lancinating pain. It is often associated with allodynia and hyperalgesia. Various nerve damaging stimuli in the peripheral or central nervous system can lead to NP. Among cancer patients, clinical studies suggest that neuropathy during cancer treatment is a predisposing event for NP^2^. Chemotherapeutic agents, including vinca alkaloids, taxanes, and platinum-based compounds, may damage peripheral nerves resulting in aberrant somatosensory processing of the peripheral or central nervous system. Surgery may also result in NP, with hypoesthesia as a significant predictive factor for the development of NP^3^. Radiation therapy may also cause NP, including brachial plexus-associated neuropathy.

NP is a major clinical problem since existing analgesics are often ineffective. First-line pharmacologic therapies include tricyclic antidepressants (eg, amitriptyline), serotonin-norepinephrine reuptake inhibitors (eg, duloxetine), and anticonvulsants (eg, gabapentin, pregabalin). However, these agents only have a modest effect. Second-line options include lidocaine patches, capsaicin high-concentration patches, and tramadol. Opiates and botulinum toxin A are typically reserved for refractory cases, but only with marginal therapeutic benefit. Studies continue to explore the mechanisms underlying NP, its treatment and management.

Genome wide association studies are an agnostic approach for exploring potential markers of disease susceptibility genes, uncovering underlying mechanisms of disease conditions and identifying novel targets for drug development. We have conducted a genome wide association (GWA) study in patients with head and neck cancer (HNSCC) to identify single nucleotide polymorphisms (SNPs) associated with neuropathy, a precursor to the development of NP.

## Results

Our study population include 1,043 patients with HNSCC, the most common type of HNC, who were treated at the Head and Neck Center at MD Anderson Cancer Center. Cases include 130 HNSCC patients with neuropathy (104 male, 26 female; mean age = 58 years; standard deviation [sd] = 10), and controls include 913 HNSCC patients without neuropathy (689 male, 224 female; mean age = 59 years; sd = 11). All patients included in this study were self-reported Caucasians. No hidden population substructures were detected based on the analysis of identity-by-state distance^4^. With the application of the standard quality control procedure, including Hardy-Weinberg proportion test, minor allele frequency (MAF) etc, we analyzed 714,494 SNPs for these 1,043 patients with HNSCC.

**Figure 1:**
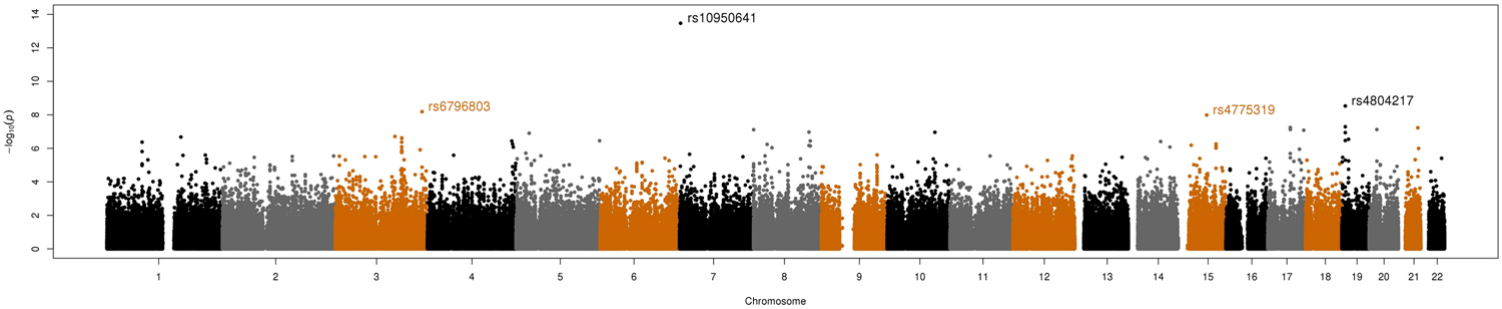
The Manhattan plot of the GWA study of neuropathy in patients with HNSCC. *P* values (as −log10 values; left y axis) were calculated based on a logistic regression model with the Fisher’s exact test.

**Figure 2:**
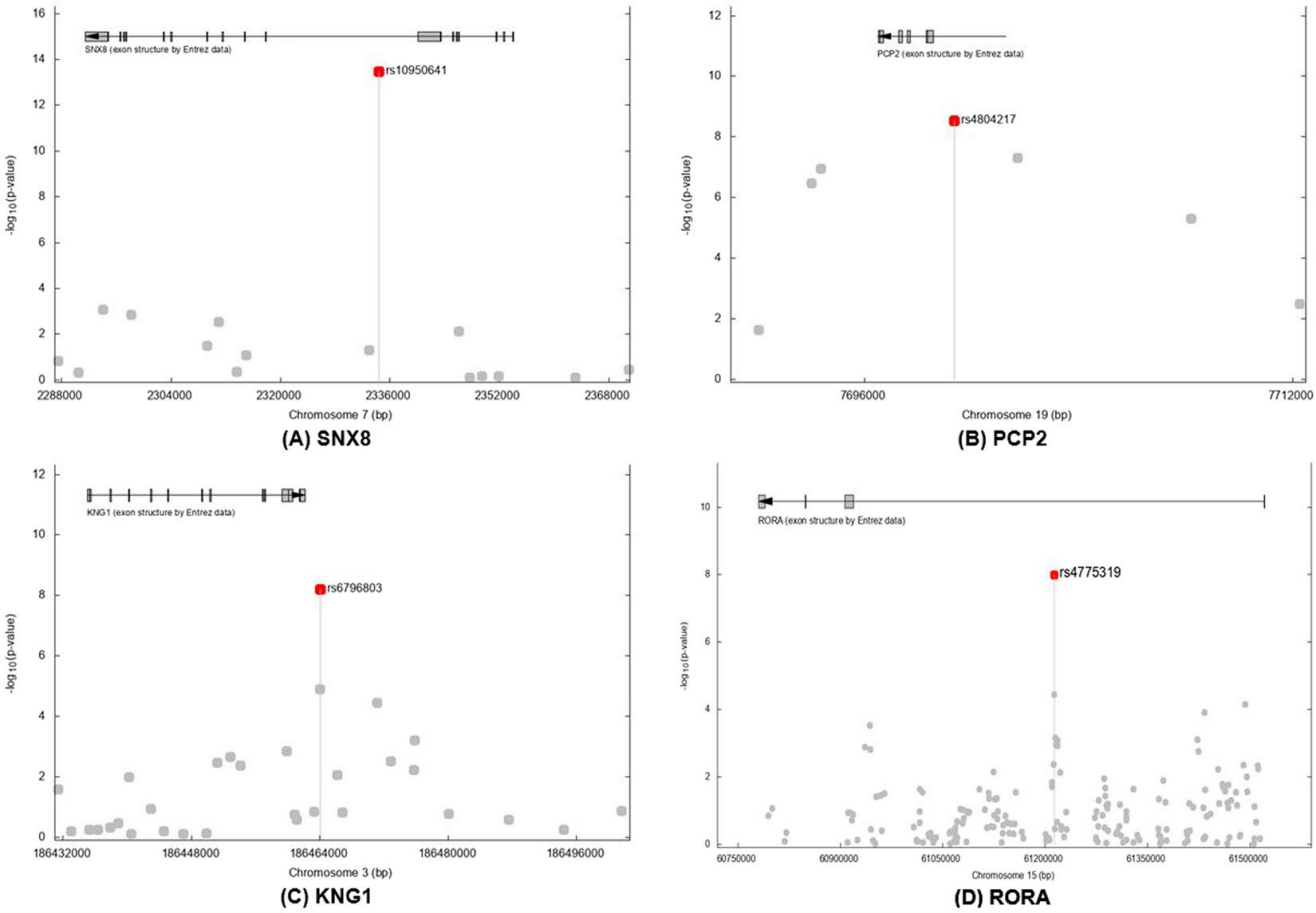
Linkage disequilibrium structure and association results for the four neuropathy-associated genomic regions. (**A**) SNX8; (**B**) PCP2; (**C**) KNG1; and (**D**) RORA. Base pair positions and genes were based on the build of the human genome GRCh37. Fisher’s exact test P values (as −log10 values; left y axis) are shown for SNPs analyzed in the GWA studies.

From the GWA analysis, we identified four SNPs representing four genomic regions that satisfied the genome-wide significance level, *P* values < 5 × 10^−08^ (Table 1). The Manhattan plot of GWA results is shown in Fig. 1 and the plots for the four genomic regions, including four significant SNPs, are shown in Fig. 2. In particular, the most significant SNP was rs10950641 (odds ratio [OR] = 2.88; 95% confidence interval [CI] = [2.19, 3.79]; *P* value = 3.39 × 10^−14^), which localizes within the gene *SNX8* (Sorting Nexin 8) on the chromosome 7 (7q22.3; 2334386 bp; Fig. 2, panel (A)). The second was rs4804217 (OR = 0.58; 95% CI = [0.48, 0.69]; *P* value = 2.95 × 10^−9^), which localizes within the gene *PCP2* (Purkinje Cell Protein 2) on the chromosome 19 (19p13.2; 7699347 bp; Fig. 2, panel (B)). Other SNPs identified included rs6796803 (OR = 0.51; 95% CI = [0.41, 0.64]; *P* value = 6.42 × 10^−9^), which localizes near gene *KNG*1 (Kininogen 1) on chromosome 3 (3q27.3; 186464107 bp; Fig. 2, panel (C)) and rs4775319 (OR = 1.59; 95% CI = [1.36, 1.87]; *P* value = 1.02 × 10^−8^), which localizes within gene *RORA* (RAR-related Orphan Receptor Alpha) on chromosome 15 (15q22.2; 61213564 bp; Fig. 2, panel (D)).

**Table 1:** Summary of results for the SNPs associated with neuropathy in patients with HNSCC.

SNP	Chr.	Gene	Location (bp)*	SNP type	MAF	Minor allele	OR [95% CI]	*P*
rs10950641	7	SNX8	2334386	Intron	0.07	A	2.88 [2.19, 3.79]	3.39E-14
rs4804217	19	PCP2	7699347	Intron	0.38	A	0.58 [0.48, 0.69]	2.95E-09
rs6796803	3	KNG1	186464107	Intergenic region, downstream	0.28	A	0.51 [0.41, 0.64]	6.42E-09
rs4775319	15	RORA	61213564	Intron	0.35	G	1.59 [1.36, 1.87]	1.02E-08

*Human annotation release 105; the build of the human genome GRCh37.

MAF: minor allele frequency; OR: odds ratio; CI: confidence interval.

## Discussion

In this study, we found that 12.5% of our patients with HNSCC received a neuropathy associated diagnosis. Using GWA study, we found four SNPs in four genes (SNX8; PCP2; KNG1; and RORA) to be statistically significantly associated with neuropathy.

Sortin Nexin 8, the most significant gene that we found to be associated with neuropathy, belongs to a family of proteins that are involved in endocytosis, endosomal sorting and signaling. Its yeast homologue (Mvp1) has been shown to be involved in endosome-to-Golgi retrieval^5^. SNX8’s role in endosomal content sorting has been implicated in the risk for neuropathologies, i.e., for late onset Alzheimer’s disease. While SNXs fundamental role is increasingly being investigated, SNXs has been recently implicated in pain. A study by Lin *et al*.^6^ showed the importance of SNXs as a critical element for glutamatergic-receptor-dependent neural plasticity and in endosomal sorting machinery in an animal model of NP. Specifically, they showed in an experimental neuropathic injury the upregulation of SNX27 and spinal vacuolar protein sorting associated protein (VPS26A); and VPSA’s recruitment and interaction with SNX27 (VPS26A-SNX27). They also showed association between VPS26A-SNX27 complex and spinal plasticity underlying NP.

Kininogen-1 (KNG1), identified as protective in this study, is a protein coding gene, which generates two different proteins, high and low molecular weight kininogens (HMWK and LMWK, respectively)^7^. Bradykinin, released from HMWK, and its related kinins are a family of small peptides which act as mediators of pain and inflammation^7,8^. KNG1 has been associated with diseases including high molecular weight kininogen deficiency and angioedema^8,9^. A study of mutant kininogen-deficient rats showed a low incidence of thermal and mechanical hyperalgesia induced by chronic nerve constriction injury suggesting that kinin were partly involved in nociceptor hypersensitivity^10^. Subsequent studies showed that kinin- receptor deficient mice have a significant reduction in paclitaxel-induced hyper-nociceptive responses compared to wild-type mice^11,12^.

Purkinje Cell Protein 2 (PCP2), identified as protective in this study, is also a protein coding gene that is expressed only in Purkinje cells of the cerebellum and in bipolar neurons of the retina, and may function as a neuron-specific modulator of intracellular signaling via G proteins^13,14^. The G_α_, G_β_ and G_γ_ form heterotrimers that transduce extracellular stimuli into intracellular responses, and the receptors coupled to G proteins constitute the largest superfamily of transmembrane receptors, ie, the G-protein-coupled receptors (GPCRs). PCP2 interacts with G_αo_^15^, which is coupled to several neuronal receptors in the central nervous system that regulate some K^+^ channels and N-type Ca^+2^ channels^16–19^. G_αo_ can also be regulated by Growth Cone-Associated Protein 43 (GAP43), also known as neuromodulin, in neurites during development^17^. Knockout of *Gap43* in mice causes derangement in neuronal pathfinding^18^; in contrast, *Gnao1* (the gene for G_αo_) knockout mice have anatomically normal appearing central nervous system but manifest motor and behavioral abnormalities and early death^16,19^. Given that μ opioid agonists act through GPCRs (e.g., *OPRM1* gene)^20^, GPCRs are therapeutic targets in pain pathways. Interestingly, a transient expression study demonstrated that transcription of *PCP*2 is activated by RAR-related orphan receptor alpha (RORA), which binds to a half-site motif of a Retinoic Acid-Related Orphan Receptor Response Element (RORE) in the promoter region^21^.

Finally, RAR-related orphan receptor alpha (RORA) is also a protein coding gene. The protein encoded by this gene has been shown to interact with NM23-1, which is the product of a tumor metastasis suppressor candidate gene^22,23^. It has been associated with diseases such as atrial tachyarrhythmia with short PR interval and trait depression^22,24–26^. Li and colleagues^27^ conducted a molecular mapping of a developing dorsal horn by microarray. The dorsal horn of the spinal cord consists of distinct lamina serving as a region for relaying somatosensory signals of touch, temperature and pain. They found for the first time, that RORA transcription factors showed a unique expression pattern in lamina II, suggesting the potential importance of RORA in pain.

**Figure 3:**
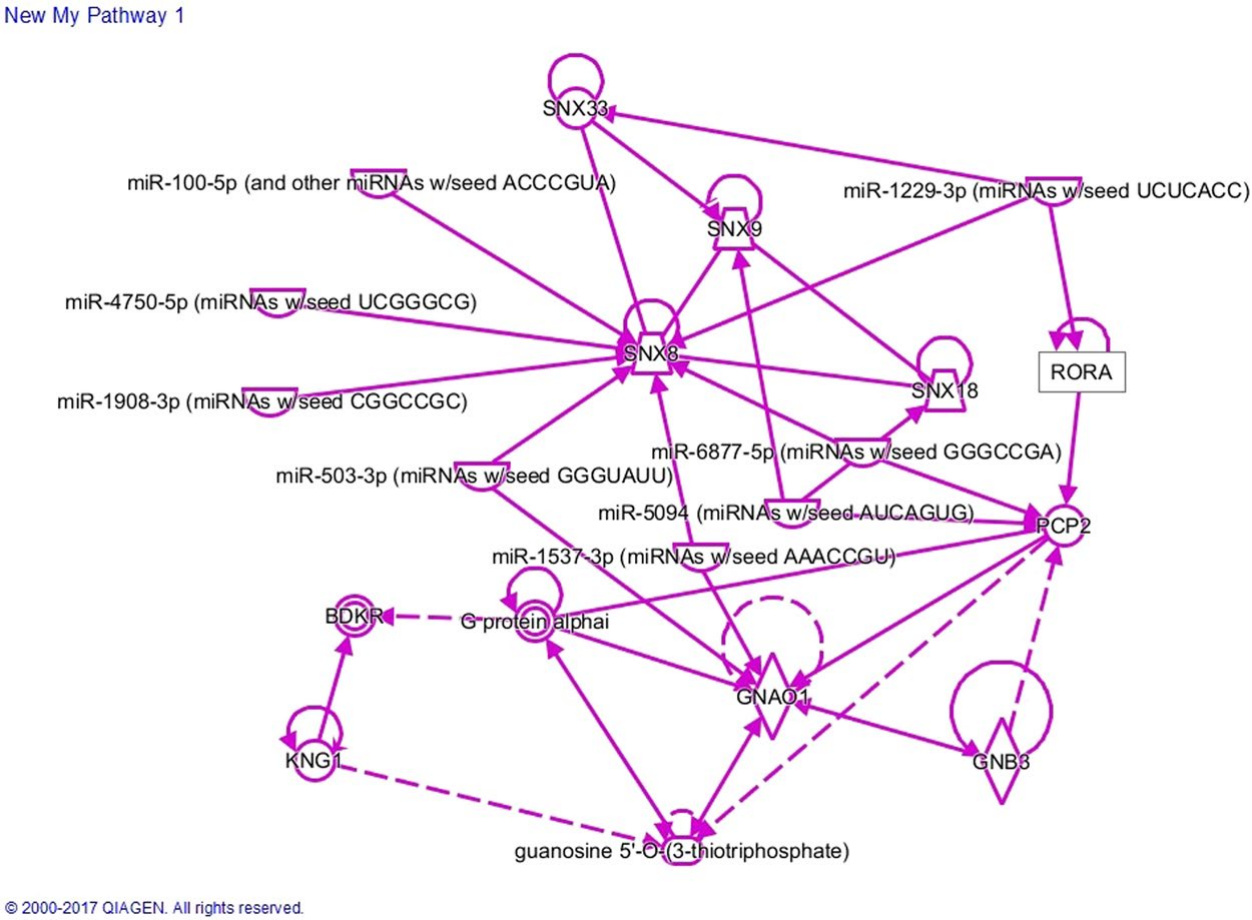
Ingenuity Pathway Analysis of SNX8, PCP2, KNG1, and RORA. Data were analyzed through the use of IPA (QIAGEN Inc., https://www.qiagenbioinformatics.com/products/ingenuitypathway-analysis).

Ingenuity Pathway Analysis (Ingenuity^®^ Systems, www.ingenuity.com) reveals that RORA, PCP2 and SNX8 can be connected in a regulatory network by microRNAs (Fig. 3). MiR-1229-39 regulates RORA and SNX8 and miR-6877-5p regulates PCP2 and SNX8. Although KNG1 belongs to a family of small peptides consisting of bradykinin and kinins which are mediators of pain and inflammation, the mechanism behind the link of this SNP near KNG1 gene with NP is unclear.

To our knowledge, this is the first GWA study to identify the importance of SNPs in SNX8, PCP2, KNG1, and RORA for neuropathy in patients with head and neck cancer. In a recent GWA study, Hertz and colleagues^28^ found that a SNP in VAC14 (rs875858) was significant for docetaxel-induced neuropathy in patients with metastatic castration-resistant prostate cancer. Conversely, a GWA study of cisplatin-induced peripheral neuropathy in testicular cancer survivors did not show significance for any of the 5.1 million SNPs but showed significance for the expression level of RPRD1B with cisplatin-induced peripheral neuropathy^29^. Other GWA studies of neuropathic pain in patients with diabetes have shown the significance of Chr8p21.3 (GFRA2)^30^ and sex-specific involvement of Chr1p35.1 (ZSCAN20-TLR12P) for females and Chr8p23.1 (HMGB1P46) for males for diabetic neuropathic pain^31^. In a recent study, Zorina-Lichtenwalter and colleagues^32^ replicated an association for two variants in IL10, which underscores the importance of neuroimmune interactions for neuropathic pain. Studies continue to explore potential genetic mechanisms underlying the development of neuropathic pain.

There are limitations to our study. Using ICD-9 and ICD-10 diagnosis for identifying patients with neuropathy may have resulted to an underestimation of its prevalence. This study should be considered exploratory and preliminary in nature, and future validation using independent data sets as well as in other cancer sites is necessary. Moreover, our study population only involved self-reported Caucasians, therefore, future GWA studies that incorporate other races or ethnicities would be important. The sample size of our study (1,043 patients with head and neck cancer, with 130 patients having neuropathy) is relatively small, implying limited statistical power. Thus, we conducted a post hoc power analysis to investigate the statistical power using the software PS: Power and Sample Size Program^33^. With a total sample size of 1,043 patients, we found that we had 80% power to detect an OR of 3.03 for a SNP‒neuropathic pain association in patients with HNC, given a genome-wide significance level of 5 × 10^−8^ to account for multiple comparisons. We might also have missed additional SNPs that could be associated with neuropathic pain, due to the limitation from our sample size. Future studies exploring the physiological effects of the identified SNPs will provide a better understanding of the biological mechanisms underlying NP and may provide insights into novel therapeutic targets for treatment and management of NP.

## Methods

### Patients

The study was approved by the Institutional Review Board at The University of Texas MD Anderson Cancer Center, and all procedures adhered to its guidelines and regulations, in accordance with the Declaration of Helsinki. All participants provided written informed consent. This study included patients with newly diagnosed and histologically confirmed HNSCC. The details of patient recruitment (including inclusion and exclusion criteria), the demographic characteristics and clinical data collection have been described in our previous study^34^.

Specifically for this study, we used the International Classification of Diseases, ninth and tenth revisions (ICD-9 and ICD-10, respectively), which is the system of codes for diagnoses and procedures in the United States, to identify neuropathy associated diagnosis among the patient cohort. We specifically searched for the following ICD-9 codes (053.13; 337.0; 337.09; 337.1; 356.4; 356.8; 356.9; 357.2; 357.3; 357.9; 377.41) and ICD-10 codes (G58.8; G58.9; G62.0; G62.2; G62.9; G63.0). Epidemiological data, such as age and sex, were also collected.

### Genotyping and Quality Controls

These procedures were previously described^34^. In brief, genomic DNA was genotyped using Illumina HumanOmniExpress-12v1 BeadChip (Illumina, San Diego, CA)^35^. Clustering and SNP calling were conducted using Illumina’s BeadStudio^36^. SNPs were considered as missing genotypes if no clusters were observed at a locus. Study genotypes were filtered and limited to autosomal SNPs with (1) call rate ≥90%; (2) MAF ≥0.05 and (3) Hardy-Weinberg proportion test *P* value ≥ 10^−6^ in control subjects^37,38^. We excluded individuals with: (1) call rates less than 95%; (2) discordant sex information and (3) duplicates. The genome-wide identity-by-state distances on SNPs^39,40^ for each pair of individuals were calculated to assess the cryptic relatedness among individuals. If two individuals have allele sharing of >80%, the one with lower call rate was excluded. We also investigate the non-Western European ancestry for all individuals by using the information from 2,502 reference samples from the 1000 Genomes Project data set (phase 3)^41^. All the quality control procedures were conducted using PLINK (v1.07)^4^. GRCh37 was the build of the human genome used for the study and the base pair (bp) locations will only match exactly with that build.

### Statistical analyses

The primary variable of interest was the binary outcome of neuropathy. The chi-squared test or Fisher’s exact test (cell count <5) was used to assess the Hardy-Weinberg proportion for each SNP^38^. A nearest neighbor cluster analysis^4,39^ was conducted to obtain the information of cluster for each individual based on genetic similarity. Specifically, with at least one case and one control in each cluster, it was obtained using pairwise population concordance at *P* value < 0.005. Associations between the neuropathy and SNPs were assessed using multivariable unconditional logistic regression, where sex, age and information of clusters were included as covariates. Significance was assessed using a Fisher’s exact test. We assumed an additive genetic model for each SNP in the association analysis. The genome-wide significance *P* value threshold of 5 × 10^−8^ was employed to account for multiple testing issue. Statistical analyses were conducted using PLINK (v1.07)^4^. Manhattan plot (Fig. 1) was generated using R software^42^. Gene Ontology (GO) information was retrieved from the Gene Ontology Annotation database^43,44^.

### Ingenuity Pathway Analysis

We used the Ingenuity Pathway Analysis (IPA, Ingenuity® Systems, www.ingenuity.com) to investigate the interactions among genes that harbor or near the SNPs (SNX8, PCP2, KNG1, and RORA) found to be significantly associated with neuropathy in our GWA study^45^. In brief, genes of interest were entered into the IPA software to build an interaction network, using the following steps: (1) The “Grow” tool was used to obtain both direct and indirect interactions of the genes of interest with other molecules. We added a maximum of 10 molecules at a time for ease of viewing and optimal performance^46^. Ingenuity Knowledge Base was used, with a limitation to only human species. (2) The “Connect” tool was used to obtain both direct and indirect connections among the genes. (3) Unconnected genes or pathways were trimmed with the “Trim” tool to improve the clarity of the network.

### Data availability

For future meta-analysis, information on all p-values are available from authors. The datasets generated during and/or analyzed during the current study are available in the dbGaP repository, phs001173.v1.p1.
